# LCN2 blockade mitigating metabolic dysregulation and redefining appetite control in type 2 diabetes

**DOI:** 10.1007/s11011-024-01454-0

**Published:** 2025-01-14

**Authors:** Cifuentes-Mendiola Saúl Ernesto, Sólis-Suarez Diana Laura, Pérez-Martínez Isaac Obed, Andrade-González Rey David, García-Gama Jahaziel Eloy, García-Hernández Ana Lilia

**Affiliations:** 1 Section of Osteimmunology and Oral Immunology, Laboratory of Dental Reseach. FES Iztacala, National Autonomous University of Mexico (UNAM), México, Mexico State México; 2 Postgraduate in Dentistry Science, National Autonomous University of Mexico (UNAM), Mexico City, Mexico; 3 Section of Sensation Neurobiology and Oral Movements, Laboratory of Dental Reseach. FES Iztacalaestigación Odontológica, National Autonomous University of Mexico (UNAM), México State México, México

**Keywords:** Type two diabetes, Lipocalin 2, Feeding pattern, Feeding control

## Abstract

LCN2 has an osteokine important for appetite regulation; in type 2 diabetes (T2D) it is not known whether appetite regulation mediated by LCN2 in the brain is altered. In this work, we focus on exploring the role of blocking LCN2 in metabolic health and appetite regulation within the central nervous system of mice with T2D. Material and methods: 4-week-old male C57BL/6 mice were used, divided into four experimental groups: intact, T2D, TD2/anti-LCN2, and T2D/IgG as isotype control. T2D was induced by low doses of streptozotocin and a high-carbohydrate diet. LCN2 blockade was performed by intraperitoneal administration of a polyclonal anti-LCN2 antibody. We analyzed metabolic parameters, food intake, feeding patterns, and serum LCN2 and leptin concentrations. In another group of intact or T2D mice, we analyzed the effect of blocking LCN2 and recombinant LCN2 on food consumption in a fasting-refeeding test and, the expression of cFOS and LCN2 in brain sections, specifically in the hypothalamus, piriform cortex, visceral area, arcuate nucleus and caudate-putamen. Results: T2D caused an increase in serum LCN2, without alterations in *Ad libitum* feeding, but with changes in the feeding pattern associated with alterations in LCN2-cFOS signalling in hypothalamic and non-hypothalamic brain regions. Blocking LCN2 improved metabolic parameters, increased *Ad libitum* feeding, and restored the feeding pattern after fasting, which is associated with enhanced LCN2 signalling in the brain. Conclusions: Blocking LCN2 restores metabolic health and normalizes the pattern of food consumption by normalizing LCN2 signalling in different brain regions.

**Graphical Abstract:**

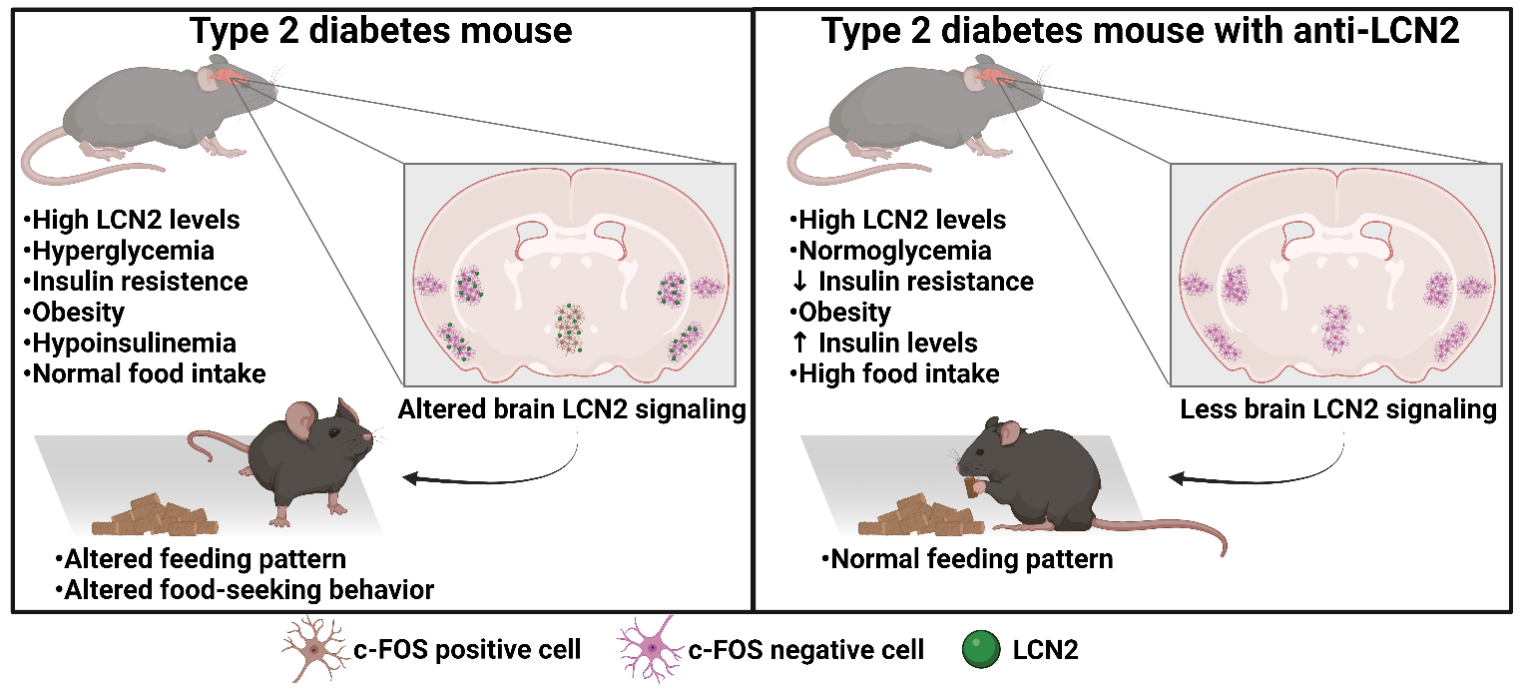

**Supplementary Information:**

The online version contains supplementary material available at 10.1007/s11011-024-01454-0.

## Introduction

Lipocalin 2 (LCN2), also identified as neutrophil gelatinase-associated lipocalin (NGAL), constitutes a 198 amino acid glycoprotein within the lipocalin superfamily-a plasma transport proteins (Jaberi et al. 2021). LCN2 is expressed in multiple cells and tissues, including neutrophils, macrophages, kidneys, lung, spleen, liver, adipose tissue, prostate, trachea, small intestine, and bone marrow (Jaberi et al. 2021). Osteoblasts within bone tissue have been described as the primary source of LCN2 (Mosialou et al. 2017).

Beyond its diverse distribution, LCN2 has multiple functions, such as innate immune defense (Flo et al. 2004), sexual reproduction, and energy metabolism through modulating food intake, insulin production, and glucose and lipid metabolism (Schröder et al. 2023). The multifaceted functions of LCN2 make it a potential molecular target for the modulation of metabolic diseases, particularly obesity and type 2 diabetes (T2D).

It has been shown in healthy, overweight, or obese mice and primates that osteoblast-derived LCN2 can cross the blood-brain barrier and bind to melanocortin receptor 4 (MC4R) on neurons in the paraventricular and ventromedial nuclei of the hypothalamus, activating the anorexigenic pathway, and suppressing appetite (Mosialou et al. 2017, 2020; Petropoulou et al. 2020); However, in T2D the role of LCN2 on appetite control is unclear and its effects on metabolism are controversial.

In this work, we focus on exploring the role of LCN2 in metabolic health and appetite regulation within the central nervous system of mice with type 2 diabetes.

## Materials and methods

### Animals

We used 4-week-old male C57BL/6 mice acquired from the Laboratorio Universitario de Bioterio del Instituto de Neurobiología (UNAM Campus Juriquilla, México). Mice were kept in a 12-light/dark hours cycle with free access to water and food. All experiments were carried out under the recommendations of the Official Mexican Standard (NOM-062-ZOO-1999), U.K. Animals (Scientific Procedures) Act, 1986, the associated guidelines, EU Directive 2010/63/EU for animal experiments and the standards of the ethics committee of the FESI, UNAM CE/FESI/112,021/1436. Mice were randomly divided into four groups: Intact, T2D, T2D/anti-LCN2, and T2D/IgG (6 mice per group, as the minimum number of specimens used in metabolic studies). At 24 weeks of age, all groups were euthanized by Pentobarbital overdose (250 mg/Kg body weight) (Dutton et al. 2019), and peripheral blood, serum, subcutaneous, and visceral adipose tissue were obtained.

### T2D induction

**Fig. 1:**
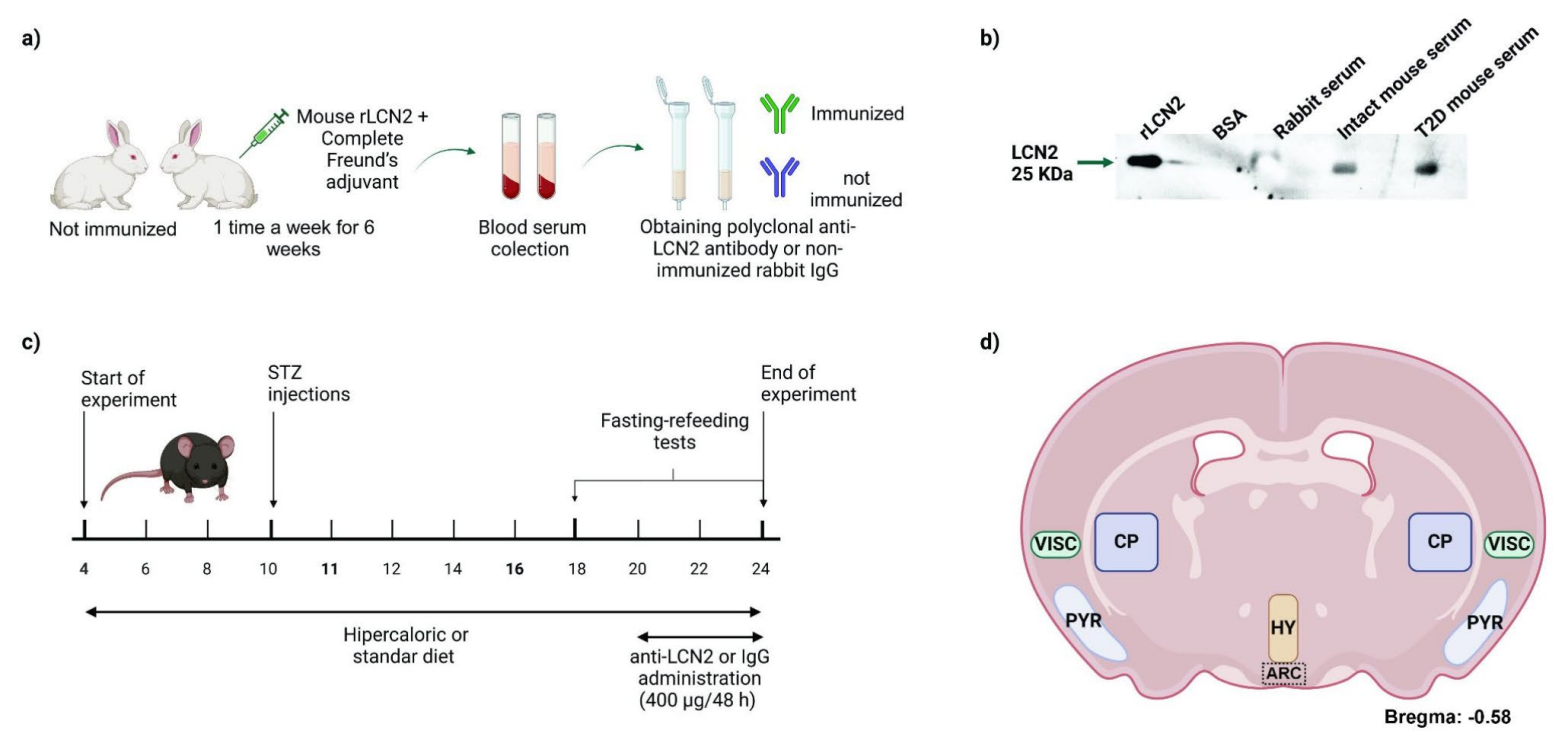
Experimental strategy. (**a**) Experimental strategy for the generation and purification of polyclonal anti-LCN2 antibodies or total IgG (isotype) produced in rabbits. (**b**) Results of the recognition and binding of the rabbit polyclonal anti-LCN2 antibody to recombinant LCN2 (rLCN2), bovine serum albumin, rabbit serum, and serum samples from intact or T2D mice by immunoblot. (**c**) Experimental strategy for T2D induction and the administration of anti-LCN2 or IgG (isotype) in C57BL/6 mice. (**d**) Illustration of a coronal section of the mouse brain showing the brain areas where the expression of cFOS and LCN2 was analyzed by IHC. rLCN2: mouse recombinant LCN2; STZ: streptozotocin; BSA: bovine serum albumin; VISC: visceral area; PYR: pyriform cortex; CP: caudate-putamen; HY: Hypothalamic paraventricular area: ARC: arcuate nucleus

T2D was induced as reported by Cifuentes-Mendiola, et al. (Cifuentes-Mendiola et al. 2023). Briefly, animals were fed with a hypercaloric diet (solid: 3.6315 kcal/g, liquid: 3.86 kcal/mL) throughout the experiment, and multiple low doses of streptozotocin (STZ, Sigma-Aldrich #S0130-1G) were injected i.p. starting at ten weeks of age daily for seven consecutive days (Day 1: 50 mg/kg of body weight, day 2–7: 25 mg/kg of body weight). Control group mice were fed with standard diet pellets (2018 S Teklad Global 18% Protein Rodent Diet; 3.1 kcal/g) and water (Fig. 1c).

### Generation and purification of anti-Lcn2 polyclonal antibody

A neutralizing anti-LCN2 polyclonal antibody was obtained per a previously reported protocol (Tarín et al. 2016). An 8-week-old New Zealand White rabbit was immunized once weekly for six weeks by intramuscular injection of 100 µg recombinant mouse LCN2 protein (rLCN2, Abcam, ab95008) diluted in 200 µL PBS and 350 µL of complete Freund’s adjuvant. After the immunization period, a blood serum was collected. IgG fraction was purified by affinity chromatography in a column charged with A-Sepharose protein (rProtein A Sepharose™ Fast Flow). The procedure was performed according to the manufacturer’s protocol. Control serum (isotype IgG) was prepared similarly from a non-immunized rabbit (Fig. 1a).

#### anti-LCN2 purified antibody functionality

Electrophoresis and western blot assays were performed to assess anti-LCN2 antibody functionality. Serum proteins were blotted onto PVDF membranes. The membranes with mice serum were incubated with purified antibody diluted at 1:500 and then incubated with horseradish peroxidase (HRP)- conjugated anti-IgG rabbit antibody diluted at 1:1000. LCN2 expression was detected in serum by chemiluminescence (SuperSignal™ West Pico PLUS Chemiluminiscent Substrate) (Fig. 1b).

### Lcn2 function inhibition

From 20 to 24 weeks of age, 400 µg of anti-LCN2 purified antibody or control rabbit IgG were administered intraperitoneally every 48 h to the T2D/anti-LCN2 and T2D/IgG group, respectively (Fig. 1c).

### Effect of LCN2 function blockade on metabolic health in T2D

Mice’s body weight was registered every third day, and abdominal circumference was measured weekly through experimentation. Subcutaneous and visceral adipose tissue were weighted upon sacrifice to determine fat mass percentage. Every 15 days, blood glucose levels were quantified after a 6-hour morning fast (8:00 AM– 2:00 PM) (Ayala et al. 2010), with an Accu-Check glucometer (One Touch Ultra Mini; Johnson & Johnson MEDICAL).

Glucose and insulin tolerance tests were performed on the mice at 18 and 23 weeks of age, with five days between each test. Briefly, the mice were fasted for 6 h (8:00 AM– 2:00 PM), then an i.p. injection of D-glucose (1.0 g/kg of body weight; Macron fine chemicals, #491212) or an i.p. shot of Aspart insulin (0.5 U/ kg of body weight; Novo Nordisk México, S.A. de CV) were administered. Blood glucose levels were monitored using a glucometer every 15 min for 90 min (Lee et al. 2018).

Per the manufacturer’s recommendations, serum insulin concentration was quantified at the time of sacrifice using the “Mouse Ins 1/ Insulin ELISA Kit” (SIGMA-ALDRICH, #0301I534).

### Effect of LCN2 on food intake in T2D

Mice were placed in individual cages with free access to a known amount of food; every third day, the mice and the remaining food were weighed to determine food and caloric intake was calculated.

### Effect of LCN2 in fasting-induced refeeding in T2D

At 18 and 24 weeks of age, fasting-refeeding tests were performed as previously described (Lu et al. 2009). Briefly, mice were fasted for 12 h, starting at 7:00 PM, and the next morning at 7:00 AM, mice were refed with a known amount of food. Food was weighed 1, 2, 4, 6, and 8 h after refeeding was initiated.

### LCN2 mechanism for appetite suppression in T2D

An additional group of intact and T2D mice (24 weeks of age, 3 mice per group) were injected i.p. with 1.5 µg of rLCN2 (Abcam, ab95008), 400 µg of anti-LCN2 or rabbit IgG after a 12 h fasting period. Mice were immediately refed with a known amount of food. Food was weighed 30, 60, and 90 min after the refeeding was initiated. All groups were euthanized by Pentobarbital overdose (250 mg/Kg body weight), after the fasting-refeeding test, and brains were extracted.

### 10. Analysis of LCN2 and cFOS expression in the brain

Mice were anesthetized with Ketamine/Xylazine (100 mg/kg, 10 mg/kg, i.p.) and transcardially perfused with 5 mL of PBS followed by 25 mL of 4% PFA in PBS. Brains were carefully extracted and fixed in 4% paraformaldehyde overnight. Brain tissue was dehydrated, clarified in xylol, and embedded in paraffin. Coronal sections were sliced at 10 μm thickness with a microtome at 6 mm deep in triplicate. The sections were rehydrated, and the antigenic recovery was carried out with a citrate buffer (pH 6) at 60° C overnight. Endogenous peroxidase and nonspecific sites were blocked, and sections were incubated with an anti-LCN2 (NGAL H-7 sc-515876 Santa Cruz Technology) or anti-cFOS (c-Fos E-8 sc166940 Santa Cruz Technology) antibody. The union was revealed with the “Super SensitiveTM Polymer HRP Kit” (Biogenex, QD400-GPN); the sections were dehydrated and mounted on microscope slides and cover-slipped.

Images of the immunostained sections were obtained with an optical microscope of the paraventricular nucleus of the hypothalamus (HY), piriform cortex (PYR), visceral area (VISC), caudate-putamen (CP) and in the arcuate nucleus of the hypothalamus (ARC) from both hemispheres (Fig. 1d). The percentage of cFOS and LCN2 expression was quantified using the free access software ImageJ.

### Statistical analysis

Data distribution and Anderson-Darling and D’Agostino & Pearson normality tests were performed. We perform multiple comparisons by one-way ANOVA test followed by a Tukey post-hoc test. In analysis with different times (weight, abdominal circumference, food intake, glucose concentration, GTT, and ITT), statistical differences were determined using a two-way ANOVA test, followed by Bonferroni’s post hoc test. All analyses were performed with GraphPad Prism 9, with a significance level of *p* < 0.05.

## Results

### LCN2 blockade reduces hyperglycemia and increases insulin production in T2D

We first determined the role of LCN2 on metabolic health and the effect of its blockade with an antibody on the severity of T2D.

**Fig. 2:**
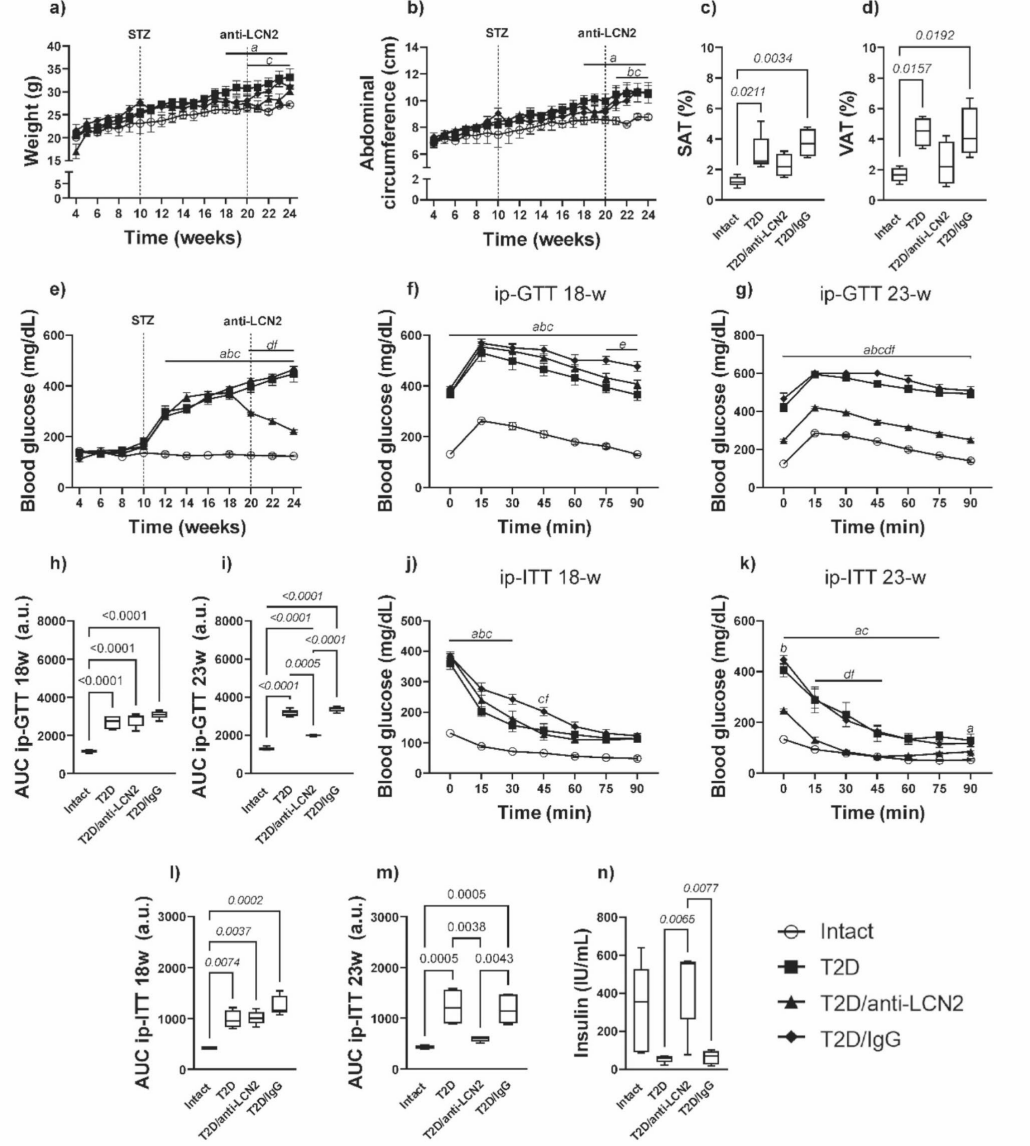
Blocking LCN2 by administering a polyclonal antibody improves metabolic health in T2D. Results of somatometric and metabolic parameters in all experimental groups. (**a**) Mice body weight (**b**) abdominal circumference (**c**) Percentage of subcutaneous adipose tissue at 24-weeks of age (**d**) Percentage of visceral adipose tissue at 24-weeks of age (**e**) Blood glucose concentrations (**f**) Glucose tolerance test (GTT) at 18-weeks of age (**g**) Glucose tolerance test (GTT) at 24-weeks of age (**h**) Area under the curve of GTT (18-weeks of age) (**i**) Area under the curve of GTT (24-weeks of age) (**j**) Insulin tolerance test (ITT) at 18-weeks of age (**k**) Insulin tolerance test (ITT) at 24-weeks of age (**l**) Area under the curve of ITT (18-weeks of age) (**m**) Area under the curve of ITT (24-weeks of age) (**n**) Serum insulin concentration. The graphs show the mean ± SEM of six mice per group ap ≤ 0.05 intact vs. T2D, bp ≤ 0.05 intact vs. T2D/anti-LCN2, cp. ≤ 0.05 intact vs. T2D/IgG, dp ≤ 0.05 T2D vs. T2D/anti-LCN2, ep ≤ 0.05 T2D vs. T2D/IgG, fp ≤ 0.05 T2D/anti-LCN2 vs. T2D/IgG. The bracket indicates the *p*-value

Mice in both the T2D and T2D/IgG (isotype control) groups exhibited central obesity, evidenced by elevated body weight, increased abdominal circumference, and the accumulation of visceral and subcutaneous adipose tissue (Fig. 2a-d). Concurrently, these groups displayed hyperglycemia, glucose intolerance, and insulin resistance, accompanied by decreased serum insulin concentration (Fig. 2e, g, i, k, m and n).

In contrast, the T2D/anti-LCN2 group, subjected to LCN2 blockade, reduced the accumulation of visceral and subcutaneous adipose tissue (Fig. 2c). Remarkably, this intervention led to a normalization of blood glucose concentration, reduction of glucose intolerance and insulin resistance, and an increase in serum insulin levels when compared to the T2D and T2D/IgG groups (Fig. 2e, g, i, k, m and n). It is pertinent to highlight that before LCN2 blockade with the antibody, at 18 weeks of age, all mice subjected to T2D induction exhibited hyperglycemia, insulin resistance, and glucose intolerance (Fig. 2e, f, h, j, and l).

### Blockade of LCN2 decreases the serum concentration of leptin, but not LCN2 in T2D mice

**Fig. 3:**
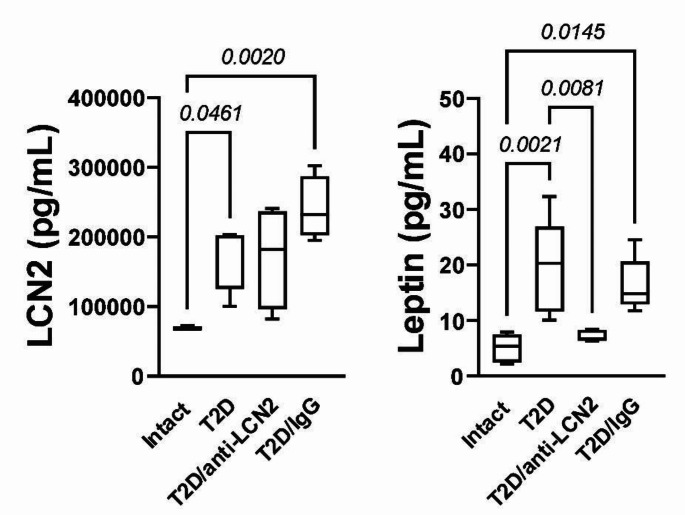
T2D increases seric LCN2 and leptin concentration while blocking LCN2 decreases only seric leptin concentration. Results of seric LCN2 and leptin concentration. The graphs show the mean ± SEM of six mice for each group. The bracket indicates p-value

We analyzed the serum concentration of LCN2 and leptin in the different experimental groups. We found that mice in the T2D and T2D/IgG groups had elevated concentrations of LCN2, and leptin compared with intact mice. However, blocking LCN2 with the antibody decreased the serum concentration of leptin but not LCN2 (Fig. 3).

### LCN2 does not suppress appetite in non-fasting T2D mice but does so after fasting

**Fig. 4:**
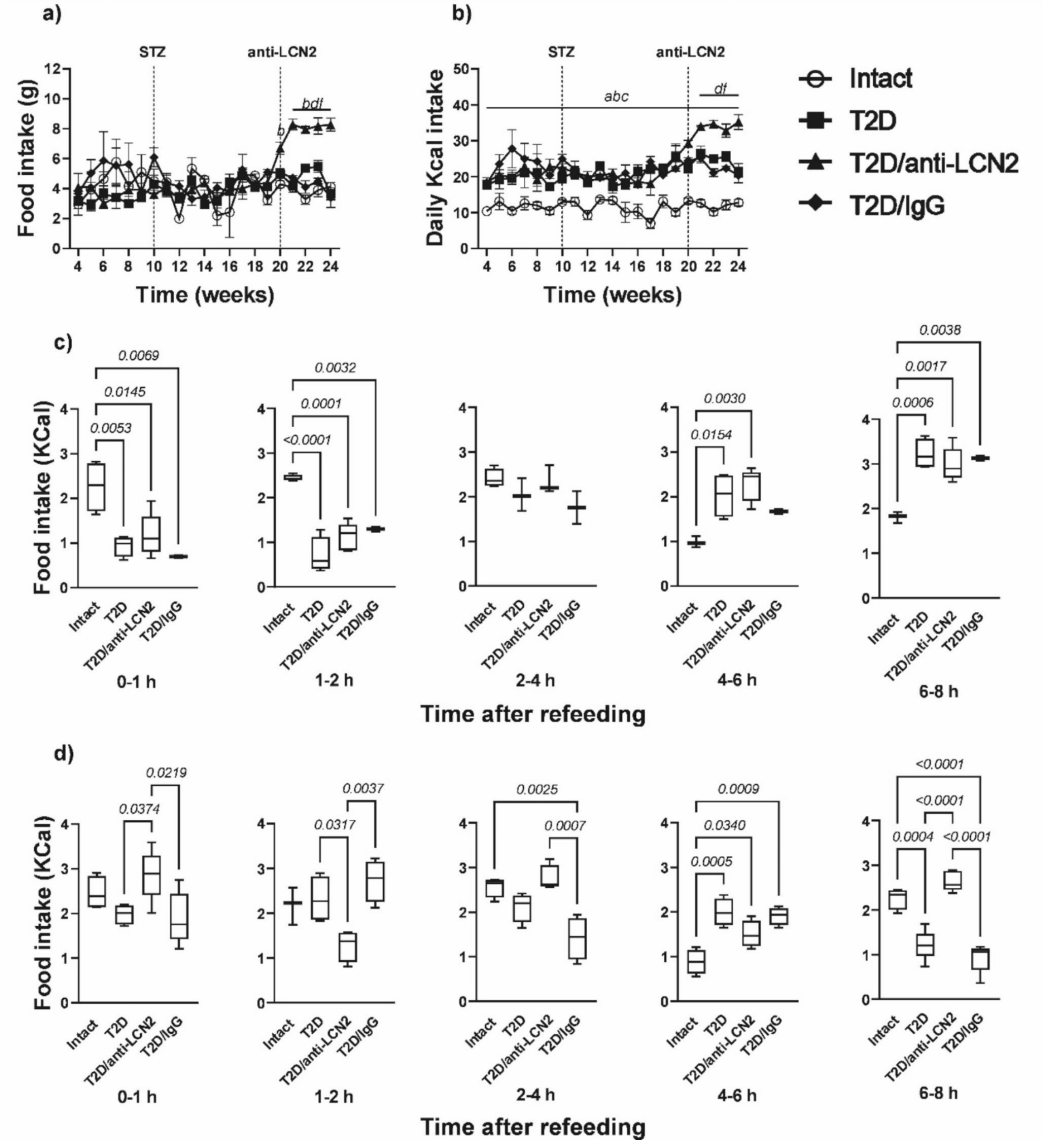
T2D mice have an altered feeding pattern, while blocking LCN2 decreases this alteration. Results of natural food consumption and eating pattern after fasting. (**a**) daily food consumption in grams throughout the experiment, (**b**) daily calorie consumption throughout the experiment, (**c**) fasting-refeeding test in calories at 18 weeks of age, and (**d**) fasting-refeeding test in calories at 24 weeks of age. The graphs show the mean ± SEM of six mice per group. ap ≤ 0.05 intact vs. T2D, bp ≤ 0.05 intact vs. T2D/anti-LCN2, cp. ≤ 0.05 intact vs. T2D/IgG, dp ≤ 0.05 T2D vs. T2D/anti-LCN2, ep ≤ 0.05 T2D vs. T2D/IgG, fp ≤ 0.05 T2D/anti-LCN2 vs. T2D/IgG. The bracket indicates *p*-value

Throughout the experiment, quantifying food intake in both grams and calories revealed comparable consumption levels between T2D and T2D/IgG mice. In contrast, the administration of the anti-LCN2 antibody in diabetic mice resulted in a significant augmentation of food consumption from the first week of antibody administration compared to the other experimental groups (Fig. 4a and b).

At 18 weeks of age, we performed a fasting-refeeding test and observed that mice in the three groups with T2D consumed less food in the first 4 h of refeeding compared to healthy mice, while 4–8 h post-refeeding they consumed more than healthy mice (Fig. 4c).

At 24 weeks of age, we performed another fasting-refeeding test. T2D and T2D/IgG mice exhibited altered feeding behaviours, contrasting with intact mice across most time points measured during food consumption assessments (Fig. 4c). In contrast, mice in the T2D/anti-LCN2 group displayed elevated food consumption compared to the T2D and T2D/IgG groups. Notably, their feeding behaviour aligned more closely with intact mice at specific intervals, particularly at one, four, and eight hours into the refeeding period (Fig. 4c).

The analysis of food consumption in grams showed a similar behaviour to the consumption in grams in all cases at 18 and 24 weeks (Supplementary Fig. 1).

### LCN2 suppresses appetite by activating hypothalamic neurons in healthy and T2D mice

To determine the mechanism by which increased LCN2 suppresses appetite in T2D mice after fasting, we performed a fasting-refeeding test in a new group of intact or T2D mice with administration of recombinant LCN2, anti-LCN2, or rabbit IgG before starting the refeeding period.

**Fig. 5:**
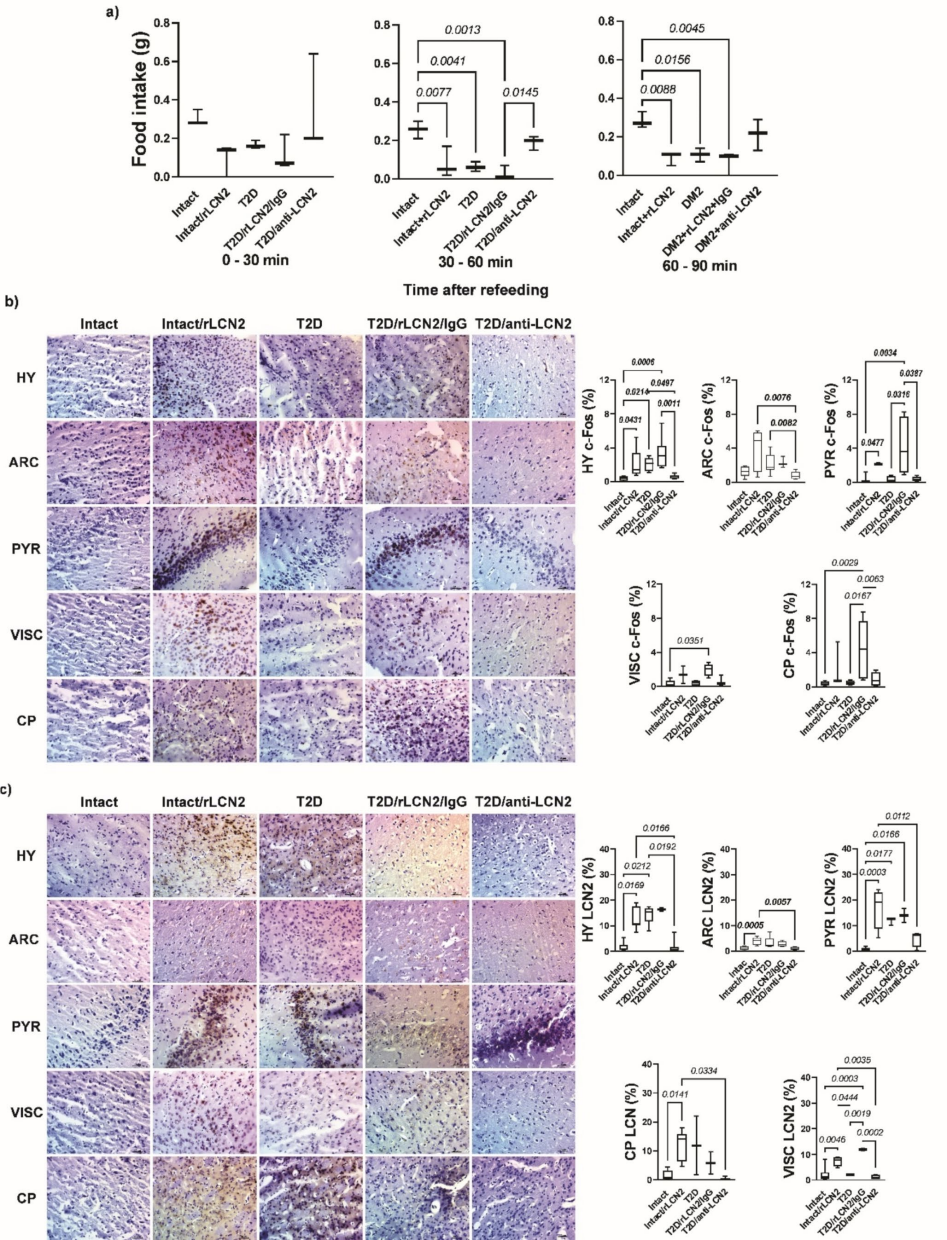
LCN2 crosses the blood-brain barrier and suppresses food intake after fasting by activating neurons in the paraventricular nucleus of the hypothalamus. Results of feeding pattern and protein expression of LCN2 and c-Fos in different brain areas after recombinant LCN2 or anti-LCN2 administration. (**a**) results of the fasting-refeed test (**b**) results of protein expression of c-Fos in the paraventricular nucleus of the hypothalamus (HY) arcuate nucleus (ARC), piriform cortex (PYR), visceral area (VISC) and caudate-putamen (CP) (**c**) protein expression of LCN2 in HY, ARC, PYR, VISC, and CP. The graphs show the mean ± SEM of three mice per group. The bracket indicates p-value

We observed that administration of recombinant LCN2 suppressed appetite in healthy and T2D mice for 90 min post-injection. Similarly, T2D-only mice showed inhibition of food consumption during the refeeding time, whereas blocking LCN2 with the antibody increased food consumption similarly to intact mice (Fig. 5a).

To understand how LCN2 controls appetite in T2D and impacts food intake in T2D, we analyzed the expression of c-Fos and LCN2 in the paraventricular area of the hypothalamus (HY) and other brain regions through IHC. In intact mice, baseline expression levels of LCN2 and cFOS were low in HY, arcuate nucleus (ARC), piriform cortex (PYR), visceral area (VISC), and caudate-putamen (CP). In contrast, the administration of recombinant LCN2 in intact mice elicited an elevation in the expression of LCN2 and cFOS in HY, ARC, PYR, VISC, and CP (Fig. 5b and c).

Mice in the T2D group had an increase in LCN2 in HY, PYR, VISC, and CP, with an increase in c-Fos only in HY and ARC. The administration of recombinant LCN2 in T2D mice had an increase in LCN2 and c-Fos in HY, ARC, PYR, VISC, and CP. In contrast, the administration of anti-LCN2 in T2D mice caused the opposite by decreasing the expression of both proteins in the same areas of the brain (Fig. 5b and c).

## Discussion

In this work, we demonstrate that LCN2 participates in the development of T2D in a mice model. Mice with T2D develop alterations in the non-fasting eating pattern, and that after fasting, LCN2 suppresses appetite by activating the hypothalamic neurons of the paraventricular nucleus and that blocking LCN2 with an antibody improves metabolic health and the pattern of food consumption in diabetes.

In T2D models featuring impaired insulin production induced by streptozotocin (STZ), administration of LCN2 has been shown to ameliorate pancreatic β cell proliferation, enhance insulin production, and reduce blood glucose levels (Mosialou et al. 2020). The role of LCN2 in the energy metabolism of type 2 diabetes is controversial and unclear. Some reports have shown that LCN2 inhibition promotes the development of diet-induced insulin resistance (Guo et al. 2010), while LCN2 administration enhances pancreatic β-cell proliferation increases insulin production and reduces insulin resistance and blood glucose levels in mice with type 2 diabetes (Mosialou et al. 2020). Our previous investigation revealed that the induction of T2D in mice led to elevated concentration of LCN2 in serum and bone tissue. Notably, this upregulation positively correlated with hyperglycemia and systemic inflammation (Cifuentes-Mendiola et al. 2022). Other preclinical and clinical studies of T2D have found an increase in circulating LCN2 associated with greater insulin resistance and hyperglycemia through promoting systemic inflammation and activation of the immune system (Moschen et al. 2017). The results presented here are consistent with previous reports indicating that LCN2 levels are elevated in T2D and this increase contributes to the development of the disease by promoting insulin resistance and hyperglycemia. (Wang et al. 2007; Park et al. 2019). Additionally, it has been reported that LCN2 inhibition protects against the development of hyperglycemia and insulin resistance in obesity (Law et al. 2010). Thus, LCN2 inhibition represents a promising therapeutic avenue for the management of T2D.

The association between the increase in LCN2 concentrations and the development and severity of T2D can be explained through the evidence that demonstrates that LCN2 stimulates the increase of TNF-a and IL-6 (Yan et al. 2007; Wu et al. 2014a; Zhao et al. 2014), cytokines that have been widely recognized as inducers of insulin resistance (Akash et al. 2013). LCN2 blockade emerges as a possible protective measure against metabolic inflammation, reinforcing its potential therapeutic efficacy in T2D.

Furthermore, our investigation unveils LCN2’s intricate role in appetite regulation. The expected anorexigenic effects of LCN2 were observed after fasting; however, the evaluation of the total food intake over a long period was not changed despite increased circulating LCN2 and leptin concentrations. This discrepancy suggests alterations in appetite regulation and food consumption patterns in T2D.

LCN2 can regulate appetite by activating the anorexigenic pathway in the hypothalamus, as has been previously demonstrated (Mosialou et al. 2017; Petropoulou et al. 2020), which in the context of T2D is relevant because alterations in food intake have been related to the development of the disease (Taylor et al. 2021); however, to date, it has been little explored.

When evaluating food intake in non-fasted mice, we observed that although T2D mice had an increase in LCN2 concentration, they consumed the same amount of food as intact mice, which was surprising because we expected an inhibition in food intake. It is worth noting that despite a presumed increase in LCN2 levels, the gram and calorie consumption of the T2D group did not change throughout the experiment. Furthermore, the administration of the antibody increased consumption, which suggested changes in the feeding pattern or resistance to LCN2 in T2D mice.

Additionally, it is very interesting to observe that sustained LCN2 blockade in the T2D group reduces serum leptin concentration. Leptin is widely known for its role in regulating appetite (Klok et al. 2007) and is typically proportional to the amount of body adipose tissue (Jéquier 2002). The decrease in leptin levels observed with the anti-LCN2 antibody administration may therefore be attributed to the reduction in body adipose tissue that we observed.

The analysis of the expression of LCN2 and c-Fos in the brain revealed that in T2D and T2D mice with recombinant LCN2, there is an increase in LCN2 and c-FOS in the paraventricular nucleus of the hypothalamus and arcuate nucleus. This increase explains the suppression of food intake in both groups and alings with the known role of these nuclei in appetite regulation Wu et al. 2014b; Valassi et al. 2008). In contrast, mice treated with the anti-LCN2 antibody showed a decrease in the expression of both proteins in these areas. This suggests that LCN2 blockade inhibits the activity of anorexigenic neurons, likely by preventing LCN2 from binding to its receptors on these neurons, thus confirming the previously reported anorexigenic function of LCN2.

Additionally, these results indicate that in T2D, LCN2 can activate neurons in the paraventricular and arcuate nuclei and induce activation of the anorexigenic pathway. However, this does not fully explain the changes in the total food intake observed over longer periods. When exploring whether LCN2 can activate other brain areas, we found intriguing results that may help explain the alterations in eating behaviour in T2D.

Interestingly, we observed that mice with T2D alone had decreased c-Fos expression in piriform cortex, visceral area, and caudate-putamen; diabetic mice with recombinant LCN2 had an increase in c-Fos expression. This is interesting because it has been reported that the activation of the piriform cortex stimulates food search behavior through smell (Ngo et al. 2022), and that the caudate-putamen participates in response to reach or mobilize to obtain food (Whishaw et al. 2007). The visceral area is known to regulate the function of the digestive tract (Azzalini et al. 2019). Therefore, it is possible that in T2D, in addition to appetite suppression mediated by the hypothalamus, the search for food and the motivation to reach for food are inhibited by LCN2, which explains the suppression in food consumption we observed in fasting states.

On the other hand, by increasing LCN2 levels through the administration of recombinant LCN2, PYR, CP, and VISC areas are activated after refeeding, which can stimulate the search for food and movement for consumption even when appetite is inhibited. This suggests that excess LCN2 may promote food consumption even when they feel full and could explain why, under *ad libitum* feeding conditions, T2D mice showed no changing consumption throughout the experiment. and the increase in food consumption after fasting in long refeeding times.

Additionally, our findings suggest that LCN2 could be related to the neuronal regulation of digestive tract function since we observed that administration with recombinant LCN2 in intact or T2D mice increases the expression of c-Fos and LCN2 in the visceral area of the brain. So, it would be interesting to investigate this in the future.

Interestingly, the systemic administration of the anti-LCN2 antibody, despite inducing greater food consumption, showed a lower expression of c-Fos and LCN2 in these three brain areas, suggesting that LCN2 can control food-seeking behavior, highlighting the potential value of conduction behavioral tests in future studies. However, the exacerbated increase in LCN2 can lead to uncontrolled eating in T2D. Therefore, it would be interesting to evaluate whether LCN2 levels fluctuate during the day and to examine the behavior of mice with elevated LCN2in the context of food-seeking in T2D.

Additionally, systemic LCN2 blockade reduces LCN2 levels in all brain areas evaluated, suggesting that the primary source of LCN2 inducing changes in the brain may be peripheral, possibly from bone tissue (Mosialou et al. 2017). However, it has also been reported that astrocytes can secrete LCN2 in response to neuroinflammation (Jung and Ryu 2023; Tan et al. 2024), a condition that can occur in T2D and has been associated with dementia (Zhang et al. 2021). Therefore, it would be important to evaluate the role of paracrine LCN2 in the brain.

## Conclusions

Although our results raise new questions and challenges to understand the role of LCN2 in T2D, our findings indicate that in T2D, there is an alteration in the regulation of appetite that LCN2 mediates and that LCN2 may also participate in complex functions of feeding-related behavior beyond hypothalamic feeding regulation. Furthermore, the increase in LCN2 engages in the development of hyperglycemia and insulin resistance, and blocking this protein can be an effective treatment for T2D.

## Electronic supplementary material

Below is the link to the electronic supplementary material.

Supplementary Material 1 (DOCX 185 KB)

## Data Availability

No datasets were generated or analysed during the current study.
