# Supplementary material for: LCN2 blockade mitigating metabolic dysregulation and redefining appetite control in type 2 diabetes
Source: Metab Brain Dis. 2025 Jan 14;40(1):97. doi: 10.1007/s11011-024-01454-0 (PMC11732943; doi:10.1007/s11011-024-01454-0)
Supplement: Supplementary file 1 — Supplementary Material 1 (DOCX 185 KB) [file 11011_2024_1454_MOESM1_ESM.docx]

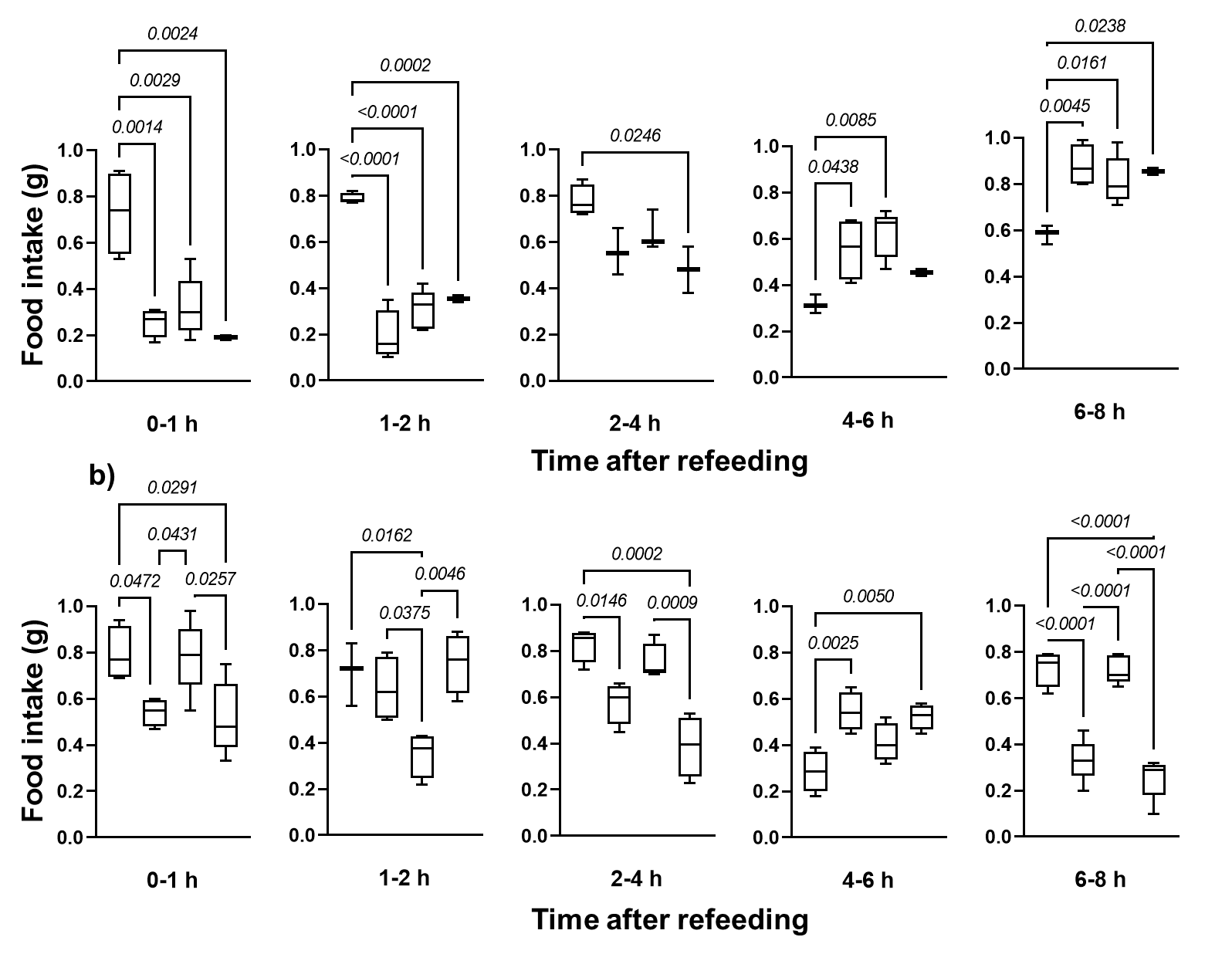


**Supplementary figure 1** T2D mice have an altered feeding calorie pattern, while blocking LCN2 decreases this alteration. Results of eating pattern after fasting in Kcal. (a) fasting-refeeding test in Kcal at 18 weeks of age, and (b) fasting-refeeding test in Kcal at 24 weeks of age. The graphs show the mean ± SEM of six mice for each group. The bracket indicates p-value
